# Analysis of Whole Blood and Urine Trace Elements in Children with Autism Spectrum Disorders and Autistic Behaviors

**DOI:** 10.1007/s12011-022-03197-4

**Published:** 2022-03-19

**Authors:** Gang Zhao, Si-jin Liu, Xin-yu Gan, Jun-ru Li, Xiao-xue Wu, Si-yan Liu, Yi-si Jin, Ke-rang Zhang, Hong-mei Wu

**Affiliations:** 1 Department of Psychiatry, First Hospital of Shanxi Medical University, Taiyuan, 030001 China; 2 Department of Child Health Care, Maternity and Child Healthcare Hospital of Nanshan District, 1 Wanxia Road, Nanshan District, Shenzhen, 518067 China; 3 Department of Nursing, Harbin Medical University in Daqing, Daqing, 163319 China; 4 Department of Rehabilitation of the Heilongjiang Province Land Reclamation Headquarters General Hospital, Harbin, 150081 China; 5 Harbin Medical University in Daqing, Daqing, 163319 Heilongjiang China; 6 Department of Rehabilitation, The Fifth Affiliated Hospital of Harbin Medical University, Daqing, 163000 China

**Keywords:** Autism spectrum disorders, Trace element, Metals, Urine analysis, Whole blood

## Abstract

The relationship between trace elements and neurological development is an emerging research focus. We performed a case–control study to explore (1) the differences of 13 trace elements chromium (Cr), manganese (Mn), cobalt (Co), zinc (Zn), arsenic (As), selenium (Se), molybdenum (Mo), cadmium (Cd), stannum (Sn), stibium (Sb), mercury (Hg), titanium (TI), and plumbum (Pb) concentration in whole blood and urine between autism spectrum disorder (ASD) children and their typical development peers, and (2) the association between the 13 trace elements and core behaviors of ASD. Thirty ASD subjects (cases) and 30 age-sex-matched healthy subjects from Baise City, Guangxi Zhuang Autonomous Region, China, were recruited. Element analysis was carried out by inductively coupled plasma-optical emission spectrometry. Autistic behaviors were assessed using Autism Behavior Checklist (ABC), Childhood Autism Rating Scale (CARS), and Children Neuropsychological and Behavior Scale (CNBS). The whole blood concentrations of Mo (*p* = 0.004), Cd (0.007), Sn (*p* = 0.003), and Pb (*p* = 0.037) were significantly higher in the ASD cases than in the controls. Moreover, Se (0.393), Hg (0.408), and Mn (− 0.373) concentrations were significantly correlated between whole blood and urine levels in ASD case subjects. There were significant correlations between whole blood Sb (0.406), Tl (0.365), Mo (− 0.4237), Mn (− 0.389), Zn (0.476), and Se (0.375) levels and core behaviors of ASD. Although the mechanism of trace element imbalance in ASD is unclear, these data demonstrate that core behaviors of ASD may be affected by certain trace elements. Further studies are recommended for exploring the mechanism of element imbalance and providing corresponding clinical treatment measures.

## Introduction

Autism spectrum disorder (ASD) is a neurodevelopmental disorder that is behaviorally characterized and defined by social communication deficits, restrictive and repetitive behavior, and interests or activities [1]. The prevalence of ASD has increased for the past few years. In the latest estimates in the USA across 11 sites, the incidence of ASD was one in 54 children aged 8 years, and was 4.3 times more likely among boys than girls, while the prevalence of ASD among children aged 6 to 12 is 0.7% in China [2–4]. The increasing prevalence of ASD has given rise to public health concerns.

Although the exact pathogenesis of ASD is unknown, it is universally believed that multiple genetic and environmental factors play significant roles in ASD development [5, 6]. In particular, it has been demonstrated that trace element imbalance may generate nervous system impairment and biological malfunctions associated with ASD [7, 8]. For example, Zn, Cu, and Se are trace elements essential for numerous physiological functions that have pivotal roles in various enzymatic processes, and hence, their biochemical regulation is vital [9]. It has been observed that antagonistic interactions between Zn and Cu can play an important role in ASD pathogenesis via modulation of the metallothionein system that induces cellular excitotoxicity [10, 11]. Other trace elements such as As, Pb, Al, and Cd play a crucial part in ASD by inducting neuroinflammation, oxidative stress, excitotoxicity, and altered essential ion homeostasis. In addition, Mn and Fe can induce neurotoxicity that can play an important role in ASD [12, 13].

There have been many studies examining essential and toxic elements in children with ASD, but the majority of these studies have concentrated on trace element levels in blood or hair [14–16]. Trace elements in blood can reflect homeostasis, whereas urine trace elements can indicate the metabolism of certain components. A minority of existing studies have examined trace element levels in urine of children with ASD and healthy peers, but these studies failed to investigate enough elements, samples, or correlations between blood and urine trace element levels [17–19].

Notably, previous research comparing ASD and typical development children has found that social communication deficits and repetitive behaviors in ASD may be related to trace element concentrations [15, 20]. Besides, a person diagnosed with autism may exhibit a variety of autistic behaviors including communication deficits, repetitive behaviors, self-injury, sleep difficulties, and dietary behavioral issues [21]. In addition, the expression of these autistic behaviors can vary widely from one individual to another [22]. Although extensive research has been carried out assessing the correlation between ASD and trace element status [23, 24], no single study exists in which the relationship between trace element status and autistic behaviors in children with ASD has been examined with Autism Behavior Checklist (ABC), Childhood Autism Rating Scale (CARS), and Children Neuropsychological and Behavior Scale (CNBS).

The purpose of this investigation is to explore the level of trace elements (Cr, Mn, Co, Zn, As, Se, Mo, Cd, Sn, Sb, Hg, TI, and Pb) in whole blood and urine samples of autistic children and compare the data with sex- and age-matched typical development children. The study also investigates possible correlations between autistic behaviors and the levels of these trace elements. This project provides an important opportunity to advance the understanding and the status of trace element nutrition in children with ASD.

## Materials and Methods

### Sample Population

The methodology applied in this study was approved by the Ethics Committee of Maternity and Child Healthcare Hospital of Nanshan District, Shenzhen. All processes were performed in line with the principles of the Declaration of Helsinki and its later amendments. Sixty participants, with age ranging from 2.5 to 5.7 years old, were enrolled from the Department of Child Health Care, Maternity and Child Healthcare Hospital of Tianyang District, Baise City, Guangxi Zhuang Autonomous Region, China. Informed consent was obtained from all the participants.

### Autism Behavior Checklist

The Autism Behavior Checklist (ABC) is a 57-item scale that aims to screen and diagnose childhood autism [25]. It is appropriate for people ranging from 2 months to 28 years of age. The scale includes five domains: sensory, relating, body/object use, language, and social/self-help.

### Childhood Autism Rating Scale

The Childhood Autism Rating Scale (CARS) is a 15-item scale that aims to make definitive diagnoses of ASD and to distinguish the severity of autistic symptomatology [26]. It is suitable for the child over the age of 2 years.

### Children Neuropsychological and Behavior Scale

The Children Neuropsychological and Behavior Scale (CNBS) is a good neurodevelopmental assessment tool that was developed by the Capital Institute of Pediatrics in China [27].

### Sampling and Trace Element Analysis

Urine and blood samples were collected early in the morning on an empty stomach. After adding 1 mL of urine sample to a 15-mL centrifuge tube, it was diluted with 9 mL of 1% nitric acid solution and then shaken with a vortex mixer. Blood samples were collected through cubital venous and stored in 4-mL lithium heparin vacuum blood collection tubes (20 international unit lithium heparin/mL). Before blood analysis, 40 μL whole blood of each sample was added to 1 mL sample diluent. The concentrations of trace elements of whole blood and urine were detected by inductively coupled plasma mass spectrometry (ICP-MS, ELAN DRCII, PerkinElmer, USA).

### Statistical Analysis

Data management and analysis were performed using Statistical Package for Social Sciences software, version 22 (SPSS Inc., Chicago, IL, USA). Normality of data distribution was assessed using the Kolmogorov–Smirnov test of normality. Normally distributed data were summarized as means and standard deviations, while non-normally distributed data were summarized as medians and the respective 25 and 75 percentile boundaries. Enumerated data were summarized as the number of cases and percentage [*n* (%)] of the total distribution. The independent *t* test (normal distribution data), the Mann–Whitney *U* test (non-normal distribution data), and the *χ*2 test (enumeration data) were used to test statistical significance between groups. All correlation analyses were performed using Spearman correlation coefficient (*r*). The level of significance was set as *p* < 0.05 for all analyses.

## Results

### Demographic Characteristics

Table 1 illustrates some of the main demographic characteristics of 60 subjects. A total of 21 boys (70%) and 9 girls (30%) participated as ASD cases while 15 boys (50%) and 15 girls (50%) participated as control subjects. The average age was 4.2 years (SD = 1.5) for cases and 3.8 years (SD = 1.3) for controls. However, there were no significant differences in sex distribution, age, weight, and height found between the case and control groups.

**Table 1:** Demographic data of ASD subjects (cases) and healthy subjects (controls)

Characteristics	Cases (*N* = 30)	Controls (*N* = 30)	*p*
Sex			
Male	21 (70)	15 (50)	0.114
Female	9 (30)	15 (50)	
Age (years)	4.2 ± 1.5	3.8 ± 1.3	0.217
Weight (kg)	16.2 ± 4.5	14.5 ± 3.8	0.125
Height (cm)	100.6 ± 13.5	98.0 ± 11.4	0.436

### Concentrations of Trace Elements in Whole Blood

We detected a total of thirty trace elements in the whole blood samples, including Cr, Mn, Co Zn, As, Se, Mo, Cd, Sn, Sb, Hg, TI, and Pb (Table 2). The results showed that the ASD cases accumulated significantly higher concentrations of Mo (*p* = 0.004), Cd (0.007), Sn (*p* = 0.003), and Pb (*p* = 0.037) than the controls (*p* < 0.05). However, there were no significant differences between ASD cases and controls for the concentrations of Cr, Mn, Co Zn, As, Se, Sb, Hg, and TI.

**Table 2:** Whole blood trace element levels (μg/L) in ASD children and healthy controls

	Control	Cases	*p*
Cr	4.265 ± 0.192	4.273 ± 0.173	0.871
Mn	12.195 (11.260–14.923)	11.495 (8.220–14.213)	0.212
Co	0.122 (0.112–0.155)	0.125 (0.111–0.166)	0.888
Zn	3881.500 (3319.250–4369.500)	4155.000 (3675.500–4816.250)	0.188
As	0.511 (0.289–0.880)	0.547 (0.408–0.758)	0.535
Se	80.826 ± 10.120	82.496 ± 14.174	0.602
Mo	0.868 (0.765–1.060)	1.172 (0.912–1.534)	0.004*
Cd	0.107 (0.081–0.169)	0.153 (0.116–0.302)	0.007*
Sn	0.609 (0.532–0.714)	0.703 (0.620–1.304)	0.003 *
Sb	2.430 (2.240–2.558)	2.360 (2.248–2.563)	0.610
Hg	0.685 (0.462–1.106)	0.796 (0.482–1.101)	0.912
Tl	0.035 (0.029–0.040)	0.033 (0.031–0.038)	0.767
Pb	11.135 (8.870–17.138)	14.960 (12.118–21.625)	0.037*

Data expressed as median (25–75) or mean ± standard deviation. *Statistical significance of group difference as assessed by the Mann–Whitney *U* test.

### Concentrations of Trace Elements in Urine

The concentrations of the trace elements Cr, Mn, Co Zn, As, Se, Mo, Cd, Sn, Sb, Hg, TI, and Pb in urine for ASD cases and controls are shown in Table 3. Notably, none of the 30 elements evaluated in this study differed significantly between ASD cases and controls (*p* > 0.05).

**Table 3:** Trace element levels (μg/L) in urine of ASD children and healthy controls

	Control	Cases	*p*
Cr	0.451 (0.100–1.320)	0.838 (0.398–1.370)	0.099
Mn	0.930 (0.376–1.678)	0.731 (0.338–1.771)	0.871
Co	0.274 (0.159–0.536)	0.272 (0.138–0.621)	0.953
Zn	435.400 (153.500–806.950)	483.300 (149.625–848.775)	0.918
As	13.870 (6.518–26.130)	17.690 (9.123–28.870)	0.171
Se	20.730 (6.935–38.765)	17.055 (8.605–38.303)	0.882
Mo	53.170 (21.390–86.058)	62.950 (29.533–85.813)	0.408
Cd	0.079 (0.036–0.154)	0.117 (0.045–0.226)	0.206
Sn	0.581 (0.214–1.276)	0.348 (0.187–0.985)	0.535
Sb	0.106 (0.069–0.153)	0.105 (0.064–0.172)	0.802
Hg	0.157 (0.020–0.250)	0.186 (0.044–0.295)	0.293
Tl	0.295 (0.120–0.399)	0.199 (0.107–0.437)	0.859
Pb	0.999 (0.587–1.519)	0.971 (0.628–1.703)	0.712

Data expressed as median (25–75) or mean ± standard deviation.

### Correlation Between Whole Blood and Urine Trace Element Levels

Table 4 shows the correlations of trace element levels between whole blood and urine samples. Correlation analysis revealed significant positive correlations between concentrations of Co (0.389), Zn (0.283), Cd (0.381), and Hg (0.469) in whole blood and urine samples of the total cohort of children. In children suffering from ASD, significant positive correlations between whole blood and urine Se (0.393) and Hg (0.408) levels were observed, whereas the association was inverse for Mn (− 0.373). In healthy children, whole blood and urine concentrations of Hg (0.593), Co (0.464), and TI (0.386) were also positively associated.

**Table 4:** Correlation between whole blood and urine trace element levels in relation to ASD and control subjects

	Total		Control		ASD	
	*r*	*p*	*r*	*p*	*r*	*p*
Cr	0.028	0.834	− 0.011	0.955	0.124	0.515
Mn	− 0.224	0.086	− 0.115	0.547	− 0.373*	0.042
Co	0.389**	0.002	0.465**	0.010	0.319	0.085
Zn	0.283*	0.028	0.264	0.159	0.328	0.077
As	0.232	0.074	0.344	0.063	0.008	0.968
Se	0.150	0.251	− 0.131	0.489	0.393*	0.032
Mo	0.046	0.729	− 0.006	0.973	− 0.029	0.878
Cd	0.318*	0.013	0.258	0.169	0.288	0.123
Sn	0.250	0.054	0.351	0.057	0.304	0.102
Sb	− 0.016	0.903	− 0.328	0.077	0.216	0.251
Hg	0.469**	0.000	0.593**	0.001	0.408*	0.025
Tl	0.204	0.117	0.386*	0.035	0.042	0.824
Pb	0.216	0.098	0.288	0.123	0.101	0.595

*r* correlation coefficient.

*Correlation is significant at *p* < 0.05.

**Correlation is significant at *p* < 0.01.

### Correlation Between Whole Blood Trace Element Concentrations and Core Symptoms of ASD Children

To test whether trace element concentrations correlate with autistic behaviors, the ABC, CARS, and CNBS were adopted to represent autistic behaviors. The results are shown in Tables 5, 6, and 7. There were two significant positive correlations detected between 2 trace elements and total scores of ABC (Sb and TS, TI and TS). Only one significant negative correlation between trace elements and test scores was found in CARS (Mo and TS). In addition, significant negative correlations were found between 2 trace elements and CNBS domains (Mn and language, TS, Mo and fine motor), and two significant positive correlations were observed (Zn and gross motor, language, Se and gross motor, TS).

**Table 5:** Spearman correlation between trace elements and ABC scores of ASD children. *r* correlation coefficient

ABC	TS	Sensory	Relating	Body/object Use	Language	Social/self-help
Cr	0.093	− 0.093	0.003	− 0.052	− 0.072	0.191
Mn	− 0.126	0.140	− 0.088	0.354	− 0.240	− 0.042
Co	0.274	0.282	− 0.099	0.202	− 0.103	0.088
Zn	− 0.023	− 0.201	0.107	− 0.078	− 0.107	0.120
As	0.056	− 0.082	− 0.139	− 0.175	− 0.311	0.360
Se	0.056	− 0.332	0.159	− 0.124	− 0.021	0.144
Mo	0.252	0.007	0.180	0.236	− 0.172	0.123
Cd	− 0.065	− 0.109	0.125	0.111	− 0.221	0.041
Sn	− 0.072	0.267	− 0.223	0.164	− 0.095	− 0.093
Sb	0.406*	0.160	− 0.148	− 0.013	0.118	0.318
Hg	0.022	− 0.188	− 0.289	0.040	− 0.034	0.181
Tl	0.365*	0.310	0.004	0.242	0.117	− 0.082
Pb	0.065	− 0.113	0.040	0.015	− 0.052	0.058

*Correlation is significant at *p* < 0.05. *TS* total scores.

**Table 6:** Spearman correlation between trace elements and CARS scores of ASD children

CARS	Total score
Cr	− 0.304
Mn	0.015
Co	0.008
Zn	− 0.032
As	− 0.334
Se	0.006
Mo	− 0.473**
Cd	0.034
Sn	− 0.009
Sb	− 0.156
Hg	− 0.068
Tl	− 0.077
Pb	0.041

*r* correlation coefficient. **Correlation is significant at *p* < 0.01. *TS* total scores.

**Table 7:** Spearman correlation between trace elements and CNBS scores of ASD children

CNBS	Adaptability	Gross motor	Fine motor	Language	Social behavior	TS
Cr	0.099	0.044	− 0.181	0.279	0.310	0.195
Mn	− 0.152	0.061	− 0.075	− 0.412*	− 0.078	− 0.389*
Co	− 0.119	− 0.183	− 0.211	− 0.117	− 0.264	− 0.355
Zn	0.032	0.396*	− 0.224	0.476**	0.096	0.286
As	0.175	− 0.025	− 0.023	0.229	0.092	0.215
Se	0.336	0.456*	− 0.275	0.354	− 0.066	0.375*
Mo	0.186	0.099	− 0.375*	− 0.086	− 0.200	− 0.226
Cd	− 0.193	0.095	− 0.187	− 0.221	− 0.008	− 0.216
Sn	0.011	0.050	− 0.074	− 0.244	− 0.172	− 0.173
Sb	− 0.185	0.104	− 0.254	0.155	− 0.066	− 0.007
Hg	− 0.059	0.220	− 0.144	0.345	0.209	0.224
Tl	0.293	− 0.210	− 0.104	0.304	− 0.138	0.129
Pb	0.002	− 0.166	0.057	− 0.281	− 0.132	− 0.268

*r* correlation coefficient. *Correlation is significant at *p* < 0.05. **Correlation is significant at *p* < 0.01. *TS* total scores.

## Discussion

The present study aims to explore possible associations between whole blood and urine levels of trace elements of autistic and healthy children. The study also attempts to investigate the possible relationship between trace elements levels and autistic behaviors in children with ASD to explore the etiology of ASD and provide a theoretical basis for treatment.

The most obvious finding that emerged from this study is that we observed significantly higher concentrations of Mo, Cd, Sn, and Pb in the whole blood of children with ASD compared to healthy children, which has not been studied in any prior whole blood study in China [28]. Mo is closely tied to sulfate/sulfite metabolism due to its essential role as a co-factor for the sulfite oxidase enzyme. Cd exposure causes the generation of ROS and genotoxicity, and evokes damage to DNA through indirect mechanisms involving induction of oxidative stress and inactivation of DNA repair proteins. Sn could induce epigenetic alterations and play a possible role in the development of children. Pb could damage the developing human brain. Children exposed to Pb are prone to experience irreversible morphological and molecular alterations of the nervous system, getting a broad spectrum of neurodevelopmental disorders such as ASD [29–31].

Significant differences in Cd and Pb concentrations between the ASD group and the control group have been reported [32, 33], but no correlation between Cd and ASD [34]. It has been reported that glia and neurons in the developing brain are prone to damage by metallic elements such as Pb which may lead to permanent neurodevelopmental damage [35]. Maria Fiore et al. demonstrated that Mo levels in hair were inversely correlated with cognitive levels in ASD [20]. In contrast to previous studies demonstrating Mo, P, and S in the urine of children with ASD were significantly lower than for their TD peers [17], no elements in urine were significantly different between the two cohorts in this study. Therefore, we think that the Mo, Cd, Sn, and Pb burden in blood is due to high exposure to these elements. Excessive metallic element exposure can impair the development of the nervous system via oxidative stress and maybe a common mechanism by which metallic elements exert their harmful effects in the brain that lead to ASD [36].

At the same time, in the ASD cohort, significant positive correlations between whole blood and urine levels of Se and Hg and a negative correlation of Mn were observed. It has been noted that there was a significant positive and negative relationship between serum and hair levels of Al and Zn, respectively, in ASD [16].

We used ABC, CARS, and CNBS to investigate the correlation between trace element concentrations and core behaviors of ASD. The risk factors (Sb and TI) showed a positive correlation with total scores of ABC. These findings add to the evidence that trace elements may contribute to the etiology of ASD. It has been shown that increased body Sb burden is associated with behavioral disorders in ASD [37]. In a similar vein, it has been reported that maternal Tl exposure at delivery can lead to negative impacts on cord serum vitamin D levels, and vitamin D deficiency has been associated with an increased risk of cognitive impairment and behavior characteristics of ASD [38–40]. This is contradictory to a previous study which showed that serum Mg and Zn levels were negatively correlated with total scores of ABC [41]. Another study of urinary toxic metals and autism-related symptoms found that Cs, Hg, and Sn tend to be correlated with ABC values [42]. Additionally, children with ASD have a reduced ability to metabolize metallic elements [43].

In contrast to Jiahui Ma et al. findings [44], our study found that Mo was negatively related to the total scores of CARS and the score of fine motor. CARS was used to assess the severity of ASD. It has been shown that vitamin/mineral supplementation (including Mo) was able to improve the severity of ASD [45]. A previous study demonstrated that there was a positive correlation between Mo and patients with mental disorders [46]. Furthermore, more studies are needed to explore whether Mo could be used as a biomarker for the diagnosis of ASD.

Deficient or excessive levels of these elements were reported to be detrimental to the neurodevelopment of children with ASD [47–49]. In our study, Se, Zn, Mo, and Mn were significantly related to CNBS scores. Several studies have suggested the association between the concentration of trace elements (Se, Zn, Mo, and Mn) and neuropsychological and behavior of ASD [15, 49, 50]. The risk factors (Se and Zn) were positively associated with certain items of CNBS, while Mo and Mn were negatively associated. These items included gross motor, language, and fine motor. Se is an essential element participating in multiple cellular processes via its structural role in selenoproteins like glutathione peroxidases (GPx). Earlier studies found that Se may be neurotoxic because of environmental overexposure and may lead to communication disorders of ASD [15, 51]. It has also been suggested that neuronal development may be damaged by deficiencies of Zn and Mo [49]. Zn is vital for brain development, antioxidant process, and synaptic transmission, closely associated with ASD-related protein. As reported in a prior study, developmental neurotoxicity caused by Mn has adverse effects on motor skills and cognitive ability [50, 52].

In the present finding, a significant increase of Cd, Sn, and Pb in the whole blood of children with ASD is noticeable; however, Cd, Sn, and Pb were not associated with core behaviors of ASD. We speculate that it is because ASD has a complex etiology including genes, environment, and diet. In addition, children with ASD have co-occurring symptoms such as seizure disorders, sleep difficulties, and gastrointestinal disturbances. Cd, Sn, and Pb may be related to co-occurring symptoms of ASD. Further studies are needed to clarify the association between trace elements and co-occurring symptoms of ASD.

## Limitations

One limitation of this case–control study was the small sample size and subjects were all from Shenzhen, China, reflecting a lack of diversity. Another limitation was dietary patterns were not assessed nor controlled between control and ASD children. It is well-known that, compared to typical developmental children, ASD children show more mealtime and eating problems, and lower diet diversity and quality [53]. Thus, it is unclear if the differences in whole blood trace elements observed in this study are due to environmental exposure and/or diet. Moreover, the current mechanisms are unknown to date; further studies are required to reveal the mechanisms of trace elements in ASD.

## Implications

This investigation is one of the first to analyze trace element concentrations in whole blood and urine of children with ASD to compare with healthy control subjects in Shenzhen, China, and to explore the correlation between core behaviors of ASD measured with CARS, ABC, and CNBS and the levels of trace elements.

## Conclusions

Our study observed that there is a significant difference in whole blood content of children with ASD and their healthy peers, with significantly higher levels of Mo, Cd, Sn, and Pb for the children with ASD. There were significant correlations between blood and urine levels of Se, Hg, and Mn in children with ASD. Sb, Tl, Mo, Mn, Zn, and Se levels in whole blood were significantly correlated with core behaviors of ASD. Prior published research has already demonstrated that the imbalance of these elements may lead to nervous system impairment and biological malfunctions associated with the pathogenesis of ASD [38, 54]. Further investigation of these elements with diet, environmental exposure, and metabolism is warranted.
